# Disturbed T Cell Signaling and Altered Th17 and Regulatory T Cell Subsets in the Pathogenesis of Systemic Lupus Erythematosus

**DOI:** 10.3389/fimmu.2015.00610

**Published:** 2015-11-30

**Authors:** Nils Rother, Johan van der Vlag

**Affiliations:** ^1^Department of Nephrology, Radboud University Medical Center, Radboud Institute of Molecular Life Sciences, Nijmegen, Netherlands

**Keywords:** systemic lupus erythematosus, T cells, autoimmunity, TCR signaling, Th17 cells, Tregs

## Abstract

Systemic lupus erythematosus (SLE) is an autoimmune disease characterized by the presence of autoantibodies against nuclear components. Circulating immune complexes of chromatin and autoantibodies deposit in various tissues leading to inflammation and tissue damage. It has been well documented that autoimmunity in SLE depends on autoreactive T cells. In this review, we summarize the literature that addresses the roles of T cell signaling, and Th17 and regulatory T cells (Tregs) in the development of SLE. T cell receptor (TCR) signaling appears to be aberrant in T cells of patients with SLE. In particular, defects in the TCRζ chain, Syk kinase, and calcium signaling molecules have been associated with SLE, which leads to hyperresponsive autoreactive T cells. Furthermore, in patients with SLE increased numbers of autoreactive Th17 cells have been documented, and Th17 cells appear to be responsible for tissue inflammation and damage. In addition, reduced numbers of Tregs as well as Tregs with an impaired regulatory function have been associated with SLE. The altered balance between the number of Tregs and Th17 cells in SLE may result from changes in the cytokine milieu that favors the development of Th17 cells over Tregs.

## Introduction

Systemic lupus erythematosus (SLE) is characterized by the presence of autoreactive antibodies against nuclear components, in particular, chromatin. Autoreactive B cells that produce pathogenic autoantibodies against nuclear components, such as double stranded DNA (dsDNA), nucleosomes, and histones, are central players in the pathogenesis of SLE (1). Autoantibodies can be detected years before the onset of clinical manifestations of the disease, which suggests a gradual loss of tolerance (2). Immune complexes of autoantibodies and chromatin deposit in various tissue, including the kidney, thereby triggering the activation of the complement system and subsequent influx of inflammatory cells (3). The vast majority of patients with SLE are female, which could be explained by a yet unknown mechanism involving the X-chromosome and/or the hormonal status (4). Prevalence of SLE varies from 20 to 150 cases per 100,000 individuals, with the highest occurrence reported in Brazil (3). To date, most patients with SLE are treated with non-specific immunosuppressive drugs and corticosteroids, which have severe side effects.

As outlined, autoantibodies in SLE are mainly directed against nuclear components, which are normally present in the nucleus inside the cell. The exposure of the immune system to released nuclear antigens in SLE patients may be explained by an aberrant apoptosis and/or impaired clearance of apoptotic cells. Apoptosis is a tightly controlled cellular program that is characterized by the breakdown of cellular components, which includes the fragmentation of chromatin. Normally, early apoptotic cells are swiftly cleared by phagocytes. However, in SLE, the process of apoptosis and/or the clearance of apoptotic material may be impaired, which could lead to the extracellular presence of chromatin triggering immune cells of the innate and adaptive immune system (5, 6). Especially, apoptotic microvesicles containing apoptosis-modified histones can be highly immunogenic through the activation of myeloid and plasmacytoid dendritic cells (DCs), and autoreactive B cells (7–12).

Normally, extracellularly present antigens ingested by antigen-presenting cells are presented in major histocompatibility complex class II molecules (MHC class II) to the T cell receptor (TCR) of CD4-positive T cells, thereby contributing to the activation of T cells. Certain lines of evidence suggest that T cells of patients with SLE are hyperresponsive compared to T cells from healthy individuals. The hyperresponsiveness of T cells in SLE may be due to changes in the signaling machinery downstream of the TCR. It has been described that autoimmunity toward chromatin in SLE depends on help of autoreactive T cells. In SLE, T cell function seems to be impaired, whereas the balance between regulatory T cells (Tregs) and Th17 cells appears to be disturbed (13–15), which will be the focus of this review.

## T Cell Activation and TCR Signaling in Healthy Individuals

Upon recognition of the antigen presented in MHC class II, the TCRs on the T cell will organize in the so-called immunological synapse. The TCR/CD3 complex, together with the co-receptor CD4, co-stimulatory molecules, and other signaling molecules are recruited to the center of the supramolecular activation cluster (SMAC) (16). The contact between the T cell and the APC is sustained through action of adhesion molecules, such as intracellular adhesion molecule 1 (ICAM-1) on the APC and lymphocyte function-associated antigen 1 (LFA-1) on the T cell. Most of the TCR/CD3 complexes and signaling molecules are present in lipid rafts already prior to engagement of the TCR. Upon binding to MHC class II, these lipid rafts serve as initiating spots of TCR signaling and fuse during the establishment of the immunological synapse (17, 18).

Antigen presented by an APC in the context of MHC class II molecules, the engagement of co-stimulatory molecules (e.g., B7 on APC and CD28 on T cells) and cytokines (e.g., IL-2) are the three signals that are required for full activation of T cells. In addition to the TCR, the co-receptor CD4 binds to MHC II. Intracellularly CD4 is associated with the lymphocyte-specific protein tyrosine kinase (Lck) that phosphorylates tyrosine residues in the immunoreceptor tyrosine-based activation motifs (ITAMs) of the TCR associated CD3 and zeta proteins (TCRζ) (19). Phosphorylated ITAMs serve as docking platforms for the Syk family tyrosine kinase Zeta-chain-associated protein kinase of 70 kDa (ZAP-70) (20). ZAP-70, in turn, phosphorylates the adaptor protein linker of activated T cells (LATs), which regulates the initiation of certain downstream-signaling pathways (21, 22). LAT can bind Ras GTP/GDP exchange factors, and thereby facilitates the activation of the Ras-mitogen-activated protein kinase (MAPK) pathway (23). LAT also serves as a docking protein for phospholipase Cγ1 (PLCγ1), which ultimately results in the activation of protein kinase C (PKC) and the release of calcium from intracellular stores (24). Activation of MAPK or PKC, and increased intracellular calcium levels leads to activation of transcription factors, such as activator protein 1 (AP-1), nuclear factor κB (NFκB), and nuclear factor of activated T cells (NFAT). Activation of the aforementioned transcription factors leads to the expression of numerous genes, an altered cytokine production, and proliferation and differentiation of T cells. T cell activation is a reversible process. Tyrosine phosphatases, for example, counteract the activity of tyrosine kinases by binding to immunoreceptor tyrosine-based inhibition motifs (ITIMs) that are present in, for example, cytotoxic T-lymphocyte antigen 4 (CTLA-4) that competes with CD28 for binding to B7 (25).

## Aberrant TCR Signaling in Patients with SLE

**Figure 1:**
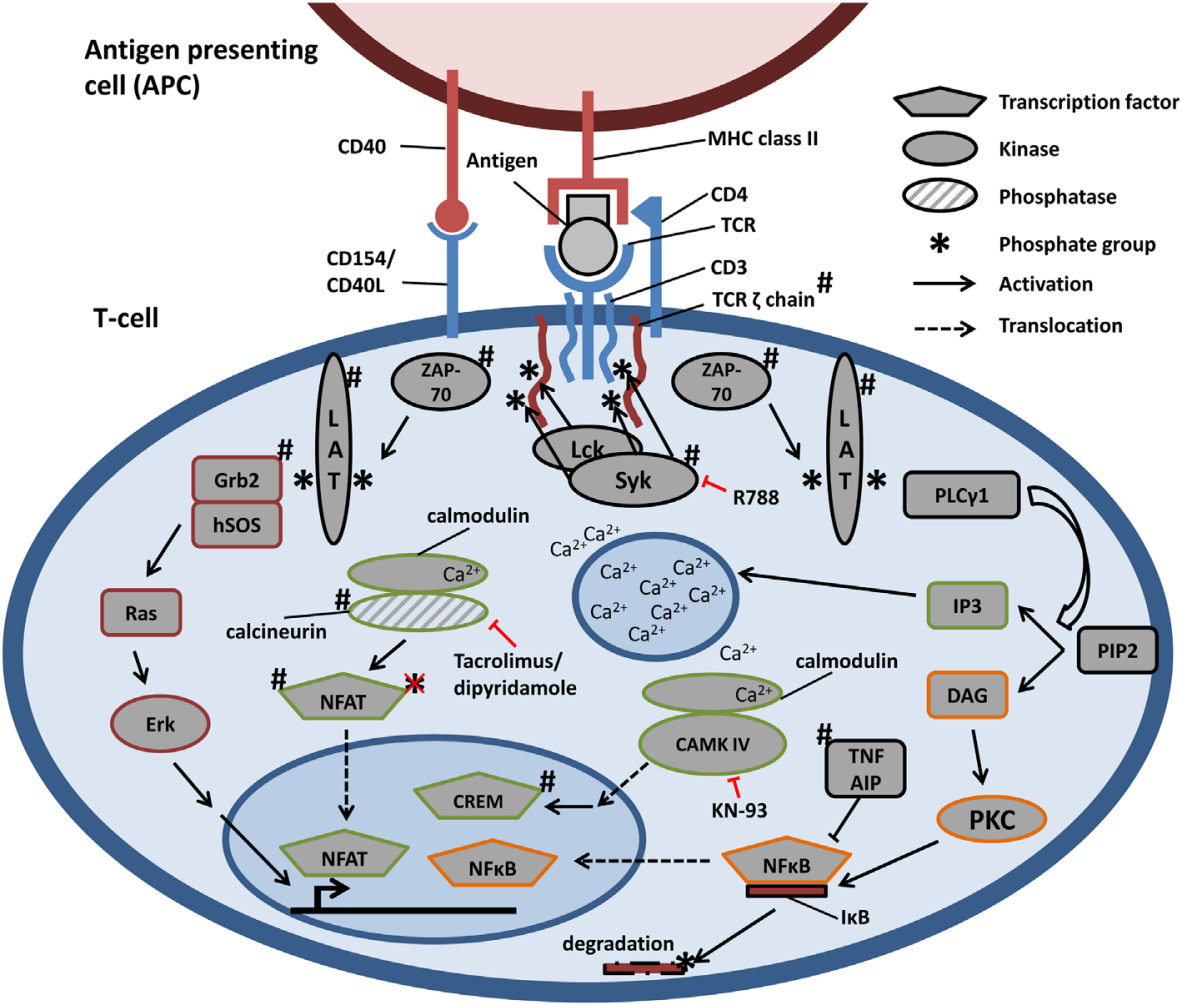
**Schematic representation of TCR-signaling pathways and aberrancies in SLE**. Engagement of TCR, through the recognition of antigen in the context of MHC class II, triggers the assembly of TCR, CD3, and TCRζ chains. TCRζ is phosphorylated and recruits ZAP-70, which in turn phosphorylates LAT. LAT serves as docking protein and phosphorylation initiates the activation of Ras–Erk, calcium-dependent, and PKC-driven signaling pathways. All signals result in the activation of transcription factors, accumulating in the nucleus and influencing gene expression. ^#^Signaling components described to be aberrantly regulated in SLE (see Table 1 and text). Therapeutic targets are depicted in red. Key: R788 is Syk inhibitor; KN-93 is CAMK IV inhibitor.

In patients with SLE, an aberrant TCR signaling has been reported, which leads to hyperresponsiveness of T cells. Changes have been found in the expression of TCRζ, the activation of intracellular spleen tyrosine kinase (Syk), calcium signaling, and various other kinase pathways (see Table 1; Figure 1) (26–28), which will be further detailed in the subsequent paragraphs.

**Table 1:** **Aberrant TCR-signaling components in SLE**.

Component	Changes in SLE T cells	Reference
**TCRζ**		
	Decreased TCRζ expression/phosphorylation	Grammatikos et al. (26), Kyttaris et al. (27), Pang et al. (29), and Takeuchi et al. (28)
	3′ UTR variants leading to instable versions of TCRζ	Chowdhury et al. (30), Gorman et al. (31), and Tsuzaka et al. (32)
**Syk**		
	Replaces canonical ZAP-70	Krishnan et al. (33) and Tanaka et al. (34)
	More potent signal transduction	Tanaka et al. (34)
	Increased expression: inability of CREMα to repress transcription	Ghosh et al. (35)
**Calcium signaling**		
	Increased recruitment of NFATc2 into the nucleus	Kyttaris et al. (36)
	→ Elevated expression of CD154	
	Enhanced nuclear import of CAMK IV	Juang et al. (37)
	→ Elevated expression of CREMα	
**Other signaling components**		
	LAT displaced from lipid rafts	Abdoel et al. (38)
	Aberrant phosphorylation of Erk	Cedeno et al. (39) and Yi et al. (40)
**Genes identified by GWAS**		
	UBASH3A	Diaz-Gallo et al. (41)
	PP2AC	Katsiari et al. (42), and Tan et al. (43)
	TNFAIP3	Graham et al. (44)
	SLAM	Morel et al. (45)

## TCRζ Expression and Function in T Cells in SLE

Expression levels of TCRζ are decreased in the majority of patients with SLE (28). In particular, TCRζ mRNA levels are lower in SLE, which may be explained by unstable mRNA variants due to polymorphisms or alternative splicing in the 3′ untranslated region (UTR) (30–32). In addition to reduced expression levels, phosphorylation of tyrosines in the ITAMs of TCRζ seems to be reduced (29). Moreover, T cells of patients with SLE are less responsive to stimulation with anti-CD3 antibodies.

The downregulation of TCRζ expression and activity, as outlined above, seems in contrast with the hyperresponsiveness of T cells in patients with SLE. However, it has been described that in T cells of patients with SLE the TCRζ can be replaced by the more potent FCγ receptor (FCγR) (46), as will be further discussed below. Furthermore, it has been shown that the inhibitory function of CTLA-4 is impaired in T cells of patients with SLE (47). Normally, CTLA-4 is associated with the tyrosine phosphatase SHP-2, which dephosphorylates TCRζ, thereby disrupting TCR signaling (48). Due to the decreased expression of TCRζ in patients with SLE, the regulatory function of CTLA-4 may be impaired. Finally, it has been suggested that impaired TCRζ signaling interferes with T cell selection processes in the thymus, which results in increased numbers of autoreactive T cells (34).

## Tyrosine Kinase Syk in T Cells in SLE

The observation that TCRζ can be replaced by FCγR in T cells of patients with SLE suggested a possible role for Syk tyrosine kinase in the pathogenesis of SLE (34), since FCγR is known to interact with tyrosine kinase Syk rather than with the canonical tyrosine kinase ZAP-70. Syk is much more potent than ZAP-70 in phosphorylating its targets, which could explain the hyperresponsiveness of T cells in SLE. Indeed, Syk expression is massively increased in T cells of patients with SLE compared to healthy individuals, whereas ZAP-70 expression levels are similar (33). Furthermore, inhibition of Syk resulted in decreased phosphorylation, actin polymerization, and calcium responses in T cells of patients with SLE, and ameliorated skin and kidney disease in a mouse model for lupus (33, 49). Importantly, Grammatikos and colleagues showed that induced expression of Syk in T cells from healthy individuals leads to expression of inflammatory factors, such as IL-21 and CD44, which could be counteracted by silencing of Syk (26).

Two underlying mechanisms leading to an increased expression of Syk in T cells of patients with SLE have been identified. First, in T cells of patients with SLE increased levels of the transcriptionfactor c-Jun drive the expression of Syk (50). Second, in SLE impaired binding of the transcriptional repressor cAMP responsive element modulator α (CREMα) in the promoter region of Syk has been shown, which is due to hypermethylation of the cAMP responsive element (35).

## Calcium Signaling in T Cells in SLE

During T cell activation PLCγ1-mediated production of inositol triphosphate (IP3) leads to increased calcium levels in the cytosol. Subsequently, calcium bound to calmodulin enables the serine/threonine phosphatase calcineurin to dephosphorylate inactive cytoplasmic phosphorylated NFAT that then translocates to the nucleus. After stimulation with either anti-CD3 antibody or PMA/ionophore, the nuclear recruitment of NFATc2 is increased in T cells of patients with SLE compared to those of healthy controls (36). Furthermore, NFATc2 shows an increased association in the promoter of the gene encoding the co-stimulatory molecule CD154 in patients with SLE compared to healthy controls, which is associated with an increased CD154 expression (36). The increased expression of CD154 most likely contributes to the hyperresponsive phenotype of T cells in SLE. Expression of NFATc1 appears to be elevated in MRL/lpr mice, an experimental model for SLE, thereby explaining the increased CD154 expression (27). Interestingly, treatment of MRL/lpr mice with dipyridamole, a drug targeting the calcineurin–NFAT pathway, reduced CD154 expression on T cells, decreased T cell dependent antibody production, and improved clinical signs of nephritis (27).

Activated calmodulin can also activate calcium/calmodulin-dependent kinase IV (CAMK IV). Activated CAMK IV translocates from the cytoplasm into the nucleus and activates transcription factors, which includes the transcriptional repressor CREMα. Nuclear import of CAMK IV is increased in T cells in SLE, which could explain the increased activation of CREMα and subsequent suppression of the gene encoding IL-2 that is negatively regulated by CREMα. Notably, IL-2 is essential for a proper development of Tregs and Th17 cells (37) and will be further detailed in paragraphs about Th17 cells and Tregs.

## Other T Cell Signaling Molecules in SLE

As outlined, LAT plays a central role in activation of T cells. It has been reported that LAT expression is decreased in patients with SLE and LAT is not found in lipid rafts compared to T cells from healthy individuals (38). Consequently, activation of the MAPK pathway is impaired, and phosphorylation of Erk1/2 is decreased in SLE compared to controls (39). Furthermore, it has been shown that the LAT-dependent coupling of Grb to human son of sevenless (hSOS), which facilitates the activation of Ras, was reduced in T cells of patients with SLE compared to T cells form healthy controls (39). Other studies showed aberrancies in peripheral T cell tolerance in SLE, i.e., induction of anergy, through sustained phosphorylation of Erk accompanied with an increased expression of the co-stimulatory molecule CD40L, thereby contributing to the persistent presence of autoreactive T cells (40).

Genome wide association studies (GWAS) revealed additional aberrancies in T cell signaling in SLE, which included a suppressor of T cell signaling called *UBASH3A* (41). Furthermore, SNPs in the *PPP2AC* gene have been associated with SLE (43). PPP2Ac expression in T cells of patients with SLE is increased, thereby reducing the production of the cytokine IL-2 that is essential for the induction of Tregs and Th17 cells (42). PP2A dephosphorylates and inactivates cAMP response element-binding protein (CREB), a transcriptional activator of IL-2 (51). Additionally, variants in the tumor necrosis factor alpha protein 3 (*TNFAIP3*) gene have been associated with SLE (44). *TNFAIP3* encodes for a zinc finger protein that negatively regulates the NFκB pathway, which is a central player in activation of immune cells and inflammatory processes (52).

## Th17 and Tregs T Cell Subsets in the Pathogenesis of SLE

T cells can be divided into multiple subsets according to their phenotype and function. Tregs are important CD4-positive T cells that function in peripheral T cell tolerance by inhibition of autoreactive T cells. Tregs can exert their tolerogenic functions via direct cell–cell contact or by the release of immunosuppressive factors, such as transforming growth factor β (TGFβ) and IL-10, whereas they are identified by the transcription factor FoxP3 (53). Reduced numbers of Tregs and impaired function of Tregs have been associated with SLE (54, 55).

Th17 cells constitute a subset of CD4-positive T cells that have been identified a decade ago. Development of Th17 cells requires TGFβ and IL-6, and they are identified by the specific transcription factor RORγt, and their characteristic production of IL-17 (56, 57). Th17 cells have been associated with chronic infection and autoimmune diseases including SLE (15).

Notably, in addition to Tregs and Th17 cells, double negative T cells, lacking both CD4 and CD8 expression, and T cells expressing the gamma and delta chain of TCR appear to play a role in the pathogenesis of SLE (58–61). However, we will focus in this review on Th17 cells and Tregs in SLE as further detailed in the subsequent paragraphs.

## Th17 Cells in SLE

Th17 cells seem to play a role in the pathogenesis of SLE. Prior to the discovery of Th17 cells, it was believed that Th1 and Th2 cells were the central players in establishing autoimmunity reactions and tissue damage. For SLE, this view changed with the identification of high levels of IL-17 and Th17 cells in patients with SLE and in mouse models for SLE (62, 63). IL-17 and Th17 cells are increasingly present in BXD2 mice that show a lupus-like autoimmune phenotype and were implicated to contribute to the formation of germinal centers and subsequent production of pathogenic antibodies (62). In other mouse models of SLE, the Ets knockout mouse (Ets^−/−^) and the New Zealand Black (NZB) × SWR F1 cross (SNF1 mice), increased levels of Th17 cells and IL-17 seem to contribute to the inflammation of kidneys, i.e., glomerulonephritis (64, 65).

Increased IL-17 production in SLE may be explained by overactive costimulation via SLAM. Polymorphisms in the cluster of SLAM encoding genes have been associated with SLE (45). Expression of *SLAMF3* and *SLAMF6* is enhanced in T cells in SLE, whereas costimulation via SLAM seems more effective compared to costimulation via CD28 in initiating the transcription of the *IL-17* encoding gene (66). Costimulation mediated by SLAMF3/SLAMF6 results in the recruitment of RORγt and NFAT to the promoter region of the *IL-1*7 encoding gene, thereby enhancing transcription of IL-17 compared to costimulation via CD28 (67). Importantly, silencing of SLAM or SLAM-associated protein (SAP) leads to a reduced production of IL-17 (66). As outlined, calcium signaling pathways, which promote the translocation of NFAT from the cytoplasm to the nucleus, are hyperactive in SLE T cells. This probably leads to higher levels of activated NFAT, which together with increased SLAM signaling drive the transcription of *IL-17* and promote Th17 development.

The transcription factor STAT3 is also involved in the development of Th17. Expression of STAT3 is increased in T cells in SLE, whereas inhibition of STAT3 leads to decreased T cell migration and delayed onset of autoimmunity in lupus prone mice (68, 69).

In addition to Th17 cells, double negative (CD4^−^, CD8^−^) T cells can produce IL-17 in SLE (58). Notably, the number of double negative T cells is increased in patients with SLE compared to healthy individuals. Taken together, an increased level of IL-17 in patients with SLE establishes an autoreactive and inflammatory environment that can lead to tissue damage.

## Regulatory T Cells in SLE

As mentioned, Tregs are important cells in establishing peripheral T cell tolerance. Therefore, the number of Tregs and function of Tregs have been studied extensively in SLE. The majority of Tregs cells are characterized by the expression of the transcription factor Foxp3 and high expression levels of the IL-2 receptor alpha chain (CD25). The development of Tregs depends on the presence of IL-2 and TGFβ. Currently, different subsets of Tregs have been described; however, we will focus in this review on Foxp3-positive cells.

The role of aberrancies in the number of Tregs and/or function of Tregs in the pathogenesis of SLE remains controversial. Some studies demonstrated a decreased number of Tregs in patients with SLE (54, 55), which could be explained by an increased susceptibility to Fas-induced cell death (55). By contrast, other reports demonstrate that the number of Tregs is similar for patients with SLE and controls (70, 71). In our opinion, the variation in outcome of different studies that address Tregs in SLE could be explained by different methods of isolation and characterization.

Regarding the suppressive function of Tregs in SLE, different claims have been published as well. Several reports demonstrate that the suppressive function of Tregs in SLE is impaired (54, 70, 72). Other reports claim that the suppressive function of Tregs in SLE is not impaired, but that autoreactive effector T cells in SLE are less susceptible to suppression by Tregs (71). However, it also has been demonstrated that Tregs from healthy controls are able to suppress effector T cells of patients with SLE (72). Taken together, Tregs seem to play a role in the pathogenesis of SLE.

## Disturbed Balance Between Tregs and Th17 Cells in SLE: A Matter of Cytokines?

**Figure 2:**
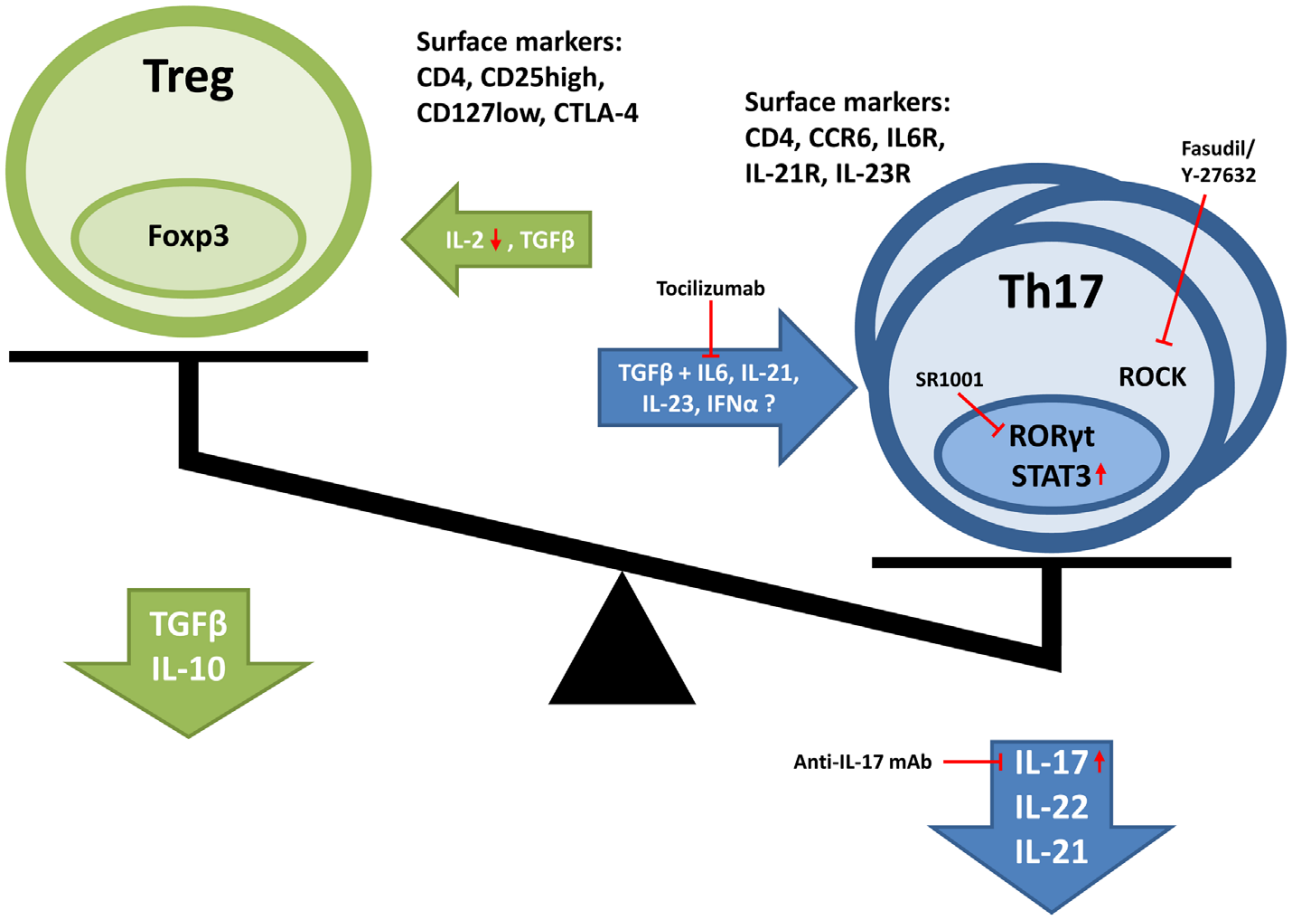
**Disturbed balance between Tregs and Th17 cells in SLE**. Cytokines important in the induction and proliferation of respective cell types are depicted. Furthermore, also characteristic surface marker, transcription factors, and produced cytokines are illustrated. Red arrows indicate changes in expression found in SLE patients. Furthermore, treatment possibilities are depicted in red.

As outlined above, there is strong evidence for a disturbed balance between Th17 cells and Tregs in patients with SLE. However, the mechanisms underlying alterations in numbers and/or function of Th17 and Tregs in SLE are only partially understood, but may involve the overall cytokine milieu (see Figure 2).

The growth factor TGFβ plays an important role in the development of both Th17 cells and Tregs. TGFβ promotes the differentiation of naïve T cells into Tregs. Furthermore, TGFβ induces the expression of *Foxp3* in γδ T cells and stimulates γδ T cells to exert suppressive functions (61). For the development of Th17 cells, the combined action of TGFβ and IL-6 is required, whereas Treg development depends on IL-2 and TGFβ. Interferon type I (IFN type I) levels, in particular IFN-α, are increased in patients with SLE (73, 74). IFN-α is primarily produced by plasmacytoid DCs (pDCs) upon recognition of nucleic acids by toll-like receptor 7 and 9 (75). IFN-α contributes to the maturation of myeloid DCs that can activate autoreactive T cells in patients with SLE. It has been suggested that IFN-α also promotes the development of Th17 cells (76). It has been shown that IFN-α triggers the production of IL-6 by myeloid DCs, whereas IL-6 is required for the development of Th17 cells (77). Moreover, it has been suggested that IFN-α impairs the suppressive function of Tregs in SLE. Importantly, in the presence of IFN-α producing DCs, Tregs of SLE patients as well as Tregs of healthy controls were not capable of suppressing T effector cells (78).

IL-2 is another cytokine that is crucial for the development of both Tregs and Th17 cells (79, 80). IL-2 is mainly produced by CD4^+^ T cells, and it is involved in the proliferation and survival of activated effector T cells. However, IL-2 or IL-2 receptor-deficient mice models show autoimmune phenotypes (81, 82), which may be explained by reduced numbers of Tregs that require IL-2 for proper development and function (83). As outlined above, production of IL-2 in patients is impaired due to the concerted action of FCγR, Syk, CREMα, and CREB (51, 84). Interestingly, increasing levels of IL-2 restore the suppressive function of Tregs in SLE when tested *in vitro* (72).

Regulatory T cells as well as Th17 cells have been shown to possess a certain degree of plasticity. It has been described that mouse Foxp3^+^ Tregs are able to transdifferentiate into Th17-like cells due to the action of IL-6 in the absence of TGFβ (85–87). Also in human, Tregs are able to adapt a Th17-like phenotype, which is accompanied with the production of IL-17 (88). It appears that Tregs that produce IL-17 can retain their suppressive function until they are triggered by IL-6 and IL-1β (89). Taken together, the cytokine milieu in patients with SLE may disturb the balance between Th17 cells and Tregs in favor of the Th17 cells, thereby explaining autoimmunity and inflammation. Dynamic changes in the cytokine milieu may transiently disturb the balance between Th17 cells and Tregs, thereby driving flares of active disease in SLE (90, 91).

## Effect of High Salt Diet on the Balance Between Tregs and Th17 Cells

Recently, it has been described that high salt intake may induce the development of Th17 cells in autoimmune diseases (92, 93). The development of Th17 cells from naïve CD4^+^ T cells was enhanced by increased sodium chloride (NaCl) concentrations in the culture medium. Furthermore, increasing the dietary intake of NaCl in an autoimmune disease mice model (experimental autoimmune encephalomyelitis, EAE) increases autoimmune features of the model. However, high salt diet in mouse models for SLE did not affect SLE disease activity (94). It has been described that NaCl activates the MAPK pathway leading to the activation of serum/glucocorticoid-regulated kinase 1 (SGK1). SGK1, in turn, suppresses the activity of Foxo1, a repressor of IL-23 expression (93). Therefore, increased NaCl concentrations result in increased production of IL-23, which contribute to the development and maintenance of Th17 cells. Increased expression levels of SGK1 have been described in patients with SLE as well (95). Interestingly, high salt intake has been correlated with a decreased effectiveness of treatment with glucocorticoids in a Chinese cohort of patients with SLE (96).

## Treatment Options Targeting T Cells in SLE

The increasing insight into the disease mechanisms of SLE allows for the development of more disease-specific drugs targeting, for example, the aberrant signaling mechanisms in T cells or the balance between Th17 cells and Tregs. Current treatment of SLE includes anti-inflammatory agents, anti-malarial drugs, glucocorticoids, and immunosuppressive medicines (3). Considering the central role of Syk in the aberrant signaling in T cells in SLE, currently specific Syk inhibitors are under development. The compound R788 inhibited the onset of kidney-related disease manifestations in mouse models for SLE (49). Furthermore, R788 was successful in the treatment of rheumatoid arthritis in a phase II clinical trial, which could encourage a clinical trial of R788 in patients with SLE as well (97).

It is well documented that inhibitors of the calcineurin–NFAT pathway, such as tacrolimus or dipyridamole decrease the production of inflammatory cytokines and reduce clinical manifestations in SLE (27). Furthermore, it has been described that blocking of CAMK IV, using the pharmacological inhibitor KN-93 suppresses production of pro-inflammatory cytokines TNFα and IFNγ, and improves proteinuria and nephritis in the MRL/lpr mouse model (98).

Targeting of co-stimulatory or adhesion receptors on T cells is another treatment option. Since CD40 is expressed at higher levels on T cells of patients with SLE compared to those of healthy controls, inhibition of either CD40 or its ligand CD40L may reduce the activation of autoreactive T cells (40). CD44 expression on T cells of patients with SLE is enhanced as well. CD44 is involved in the adhesion of T cells and has been associated with increased cell migration toward the kidneys, thereby contributing to inflammation and damage of the kidney (99). Engagement of CD44 leads to the activation of Rho kinase (ROCK) and production of IL-17. Therefore, inhibitors of ROCK could disrupt CD44-mediated signaling in T cells in SLE (100). Abatacept is a CTLA-4 immunoglobulin fusion protein that is used to compete with CD28 for binding to B7 proteins, thereby reducing activation of autoreactive T cells.

Restoration of the balance between Th17 cells and Tregs in patients with SLE could be achieved by manipulation of the cytokine milieu in such a way that the development of Tregs is favored over Th17 cells. A strategy could include the blocking of IL-17 or IL-23, cytokines important for the development of Th17 cells. However, blocking IL-17 alone showed a worsening of the disease phenotype in mouse models (101, 102). More encouraging is the blocking of the IL-6 receptor using a monoclonal antibody, tocilizumab, which showed promising results in clinical phase I studies (103). Moreover, the inhibition of the main transcription factor involved in the development of Th17 cells, RORγt, using a synthetic molecule (SR1001) also appears to be beneficial in blocking the differentiation of Th17 cells (104).

## Concluding Remarks

We summarized multiple mechanism and pathways impaired in T cells from patients with SLE. A note of caution should be made, since in SLE, there is no general and/or complete picture regarding its pathogenesis when comparing individual patients. So, not all aberrancies in T cell signaling and disturbances in Th17 cell/Tregs as described in this review are present in all patients with SLE. Nevertheless, the signaling cascades emerging from the TCR and ultimately leading to changes in the expression of numerous genes play a key role in the phenotype of T cells in SLE. In general, aberrancies in TCR signaling lead to a hyperresponsive state of T cells in SLE. Moreover, aberrancies in TCR signaling may affect selection processes in the thymus, thereby impairing central tolerance. Small molecule inhibitors specific for certain key signaling molecules (e.g., Syk and CAMK IV) have been developed and show promising results in pre-clinical as well as in clinical studies. The interplay between Tregs, Th17 cells, and other cell subsets is complex and only in part understood. Restoring a healthy balance between Tregs and Th17 cells in patients with SLE is not a straight forward therapeutic option yet, but an interesting research goal that may be translated into clinical practice in a later phase.

## Conflict of Interest Statement

The authors declare that the research was conducted in the absence of any commercial or financial relationships that could be construed as a potential conflict of interest.

## References

[1] Mohan C Adams S Stanik V Datta SK. Nucleosome: a major immunogen for pathogenic autoantibody-inducing T cells of lupus. J Exp Med (1993) 177:1367–81. 10.1084/jem.177.5.1367 8478612 PMC2191002

[2] Arbuckle MR McClain MT Rubertone MV Scofield RH Dennis GJ James JA Development of autoantibodies before the clinical onset of systemic lupus erythematosus. N Engl J Med (2003) 349:1526–33. 10.1056/NEJMoa021933 14561795

[3] Tsokos GC Systemic lupus erythematosus. N Engl J Med (2011) 365:2110–21. 10.1056/NEJMra1100359 22129255

[4] Weckerle CE Niewold TB. The unexplained female predominance of systemic lupus erythematosus: clues from genetic and cytokine studies. Clin Rev Allergy Immunol (2011) 40:42–9. 10.1007/s12016-009-8192-4 20063186 PMC2891868

[5] Munoz LE van Bavel C Franz S Berden J Herrmann M van der Vlag J. Apoptosis in the pathogenesis of systemic lupus erythematosus. Lupus (2008) 17:371–5. 10.1177/0961203308089990 18490410

[6] Pieterse E van der Vlag J. Breaking immunological tolerance in systemic lupus erythematosus. Front Immunol (2014) 5:164. 10.3389/fimmu.2014.00164 24782867 PMC3988363

[7] Dieker J Hilbrands L Thielen A Dijkman H Berden JH van der Vlag J Enhanced activation of dendritic cells by autologous apoptotic microvesicles in MRL/lpr mice. Arthritis Res Ther (2015) 17:103 10.1186/s13075-015-0617-2 25886192 PMC4422546

[8] Dieker J Tel J Pieterse E Thielen A Rother N Bakker M Circulating apoptotic microparticles in SLE patients drive the activation of DC subsets and prime neutrophils for NETosis. Arthritis Rheumatol (2015). 10.1002/art.39417 26360137

[9] Dieker JW Fransen JH van Bavel CC Briand JP Jacobs CW Muller S Apoptosis-induced acetylation of histones is pathogenic in systemic lupus erythematosus. Arthritis Rheum (2007) 56:1921–33. 10.1002/art.22646 17530637

[10] Price JV Tangsombatvisit S Xu G Yu J Levy D Baechler EC On silico peptide microarrays for high-resolution mapping of antibody epitopes and diverse protein-protein interactions. Nat Med (2012) 18:1434–40. 10.1038/nm.2913 22902875 PMC3491111

[11] van Bavel CC Dieker JW Kroeze Y Tamboer WP Voll R Muller S Apoptosis-induced histone H3 methylation is targeted by autoantibodies in systemic lupus erythematosus. Ann Rheum Dis (2011) 70:201–7. 10.1136/ard.2010.129320 20699234

[12] van Bavel CC Dieker JW Tamboer WP van der Vlag J Berden JH. Lupus-derived monoclonal autoantibodies against apoptotic chromatin recognize acetylated conformational epitopes. Mol Immunol (2010) 48:248–56. 10.1016/j.molimm.2010.08.003 20817300

[13] Alunno A Bartoloni E Bistoni O Nocentini G Ronchetti S Caterbi S Balance between regulatory T and Th17 cells in systemic lupus erythematosus: the old and the new. Clin Dev Immunol (2012) 2012:823085. 10.1155/2012/823085 22761634 PMC3386568

[14] Crispin JC Kyttaris VC Terhorst C Tsokos GC T cells as therapeutic targets in SLE. Nat Rev Rheumatol (2010) 6:317–25. 10.1038/nrrheum.2010.60 20458333 PMC2924434

[15] Garrett-Sinha LA John S Gaffen SL. IL-17 and the Th17 lineage in systemic lupus erythematosus. Curr Opin Rheumatol (2008) 20:519–25. 10.1097/BOR.0b013e328304b6b5 18698171

[16] Monks CR Freiberg BA Kupfer H Sciaky N Kupfer A. Three-dimensional segregation of supramolecular activation clusters in T cells. Nature (1998) 395:82–6. 10.1038/25764 9738502

[17] Campi G Varma R Dustin ML. Actin and agonist MHC-peptide complex-dependent T cell receptor microclusters as scaffolds for signaling. J Exp Med (2005) 202:1031–6. 10.1084/jem.20051182 16216891 PMC1373686

[18] Yokosuka T Sakata-Sogawa K Kobayashi W Hiroshima M Hashimoto-Tane A Tokunaga M Newly generated T cell receptor microclusters initiate and sustain T cell activation by recruitment of Zap70 and SLP-76. Nat Immunol (2005) 6:1253–62. 10.1038/ni1272 16273097

[19] Kane LP Lin J Weiss A. Signal transduction by the TCR for antigen. Curr Opin Immunol (2000) 12:242–9. 10.1016/S0952-7915(00)00083-2 10781399

[20] Chan AC Iwashima M Turck CW Weiss A. ZAP-70: a 70 kd protein-tyrosine kinase that associates with the TCR zeta chain. Cell (1992) 71:649–62. 10.1016/0092-8674(92)90598-7 1423621

[21] Rossy J Williamson DJ Benzing C Gaus K. The integration of signaling and the spatial organization of the T cell synapse. Front Immunol (2012) 3:352. 10.3389/fimmu.2012.00352 23189081 PMC3504718

[22] Samelson LE. Signal transduction mediated by the T cell antigen receptor: the role of adapter proteins. Annu Rev Immunol (2002) 20:371–94. 10.1146/annurev.immunol.20.092601.111357 11861607

[23] Rincon M Flavell RA Davis RJ Signal transduction by MAP kinases in T lymphocytes. Oncogene (2001) 20:2490–7. 10.1038/sj.onc.1204382 11402343

[24] Oh-hora M Rao A. Calcium signaling in lymphocytes. Curr Opin Immunol (2008) 20:250–8. 10.1016/j.coi.2008.04.004 18515054 PMC2574011

[25] Acuto O Michel F CD28-mediated co-stimulation: a quantitative support for TCR signalling. Nat Rev Immunol (2003) 3:939–51. 10.1038/nri1248 14647476

[26] Grammatikos AP Ghosh D Devlin A Kyttaris VC Tsokos GC. Spleen tyrosine kinase (Syk) regulates systemic lupus erythematosus (SLE) T cell signaling. PLoS One (2013) 8:e74550. 10.1371/journal.pone.0074550 24013589 PMC3754955

[27] Kyttaris VC Zhang Z Kampagianni O Tsokos GC. Calcium signaling in systemic lupus erythematosus T cells: a treatment target. Arthritis Rheum (2011) 63:2058–66. 10.1002/art.30353 21437870 PMC3128171

[28] Takeuchi T Suzuki K Kondo T Yoshimoto K Tsuzaka K CD3 zeta defects in systemic lupus erythematosus. Ann Rheum Dis (2012) 71(Suppl 2):i78–81. 10.1136/annrheumdis-2011-200641 22460144

[29] Pang M Setoyama Y Tsuzaka K Yoshimoto K Amano K Abe T Defective expression and tyrosine phosphorylation of the T cell receptor zeta chain in peripheral blood T cells from systemic lupus erythematosus patients. Clin Exp Immunol (2002) 129:160–8. 10.1046/j.1365-2249.2002.01833.x 12100036 PMC1906428

[30] Chowdhury B Tsokos CG Krishnan S Robertson J Fisher CU Warke RG Decreased stability and translation of T cell receptor zeta mRNA with an alternatively spliced 3’-untranslated region contribute to zeta chain down-regulation in patients with systemic lupus erythematosus. J Biol Chem (2005) 280:18959–66. 10.1074/jbc.M501048200 15743765

[31] Gorman CL Russell AI Zhang Z Cunninghame Graham D Cope AP Vyse TJ. Polymorphisms in the CD3Z gene influence TCRzeta expression in systemic lupus erythematosus patients and healthy controls. J Immunol (2008) 180:1060–70. 10.4049/jimmunol.180.2.1060 18178846

[32] Tsuzaka K Fukuhara I Setoyama Y Yoshimoto K Suzuki K Abe T TCR zeta mRNA with an alternatively spliced 3’-untranslated region detected in systemic lupus erythematosus patients leads to the down-regulation of TCR zeta and TCR/CD3 complex. J Immunol (2003) 171:2496–503. 10.4049/jimmunol.171.5.2496 12928398

[33] Krishnan S Juang YT Chowdhury B Magilavy A Fisher CU Nguyen H Differential expression and molecular associations of Syk in systemic lupus erythematosus T cells. J Immunol (2008) 181:8145–52. 10.4049/jimmunol.181.11.8145 19018007 PMC2586973

[34] Tanaka S Maeda S Hashimoto M Fujimori C Ito Y Teradaira S Graded attenuation of TCR signaling elicits distinct autoimmune diseases by altering thymic T cell selection and regulatory T cell function. J Immunol (2010) 185:2295–305. 10.4049/jimmunol.1000848 20644168

[35] Ghosh D Kis-Toth K Juang YT Tsokos GC CREMalpha suppresses spleen tyrosine kinase expression in normal but not systemic lupus erythematosus T cells. Arthritis Rheum (2012) 64:799–807. 10.1002/art.33375 21953500 PMC3250560

[36] Kyttaris VC Wang Y Juang YT Weinstein A Tsokos GC. Increased levels of NF-ATc2 differentially regulate CD154 and IL-2 genes in T cells from patients with systemic lupus erythematosus. J Immunol (2007) 178:1960–6. 10.4049/jimmunol.178.3.1960 17237447

[37] Juang YT Wang Y Solomou EE Li Y Mawrin C Tenbrock K Systemic lupus erythematosus serum IgG increases CREM binding to the IL-2 promoter and suppresses IL-2 production through CaMKIV. J Clin Invest (2005) 115:996–1005. 10.1172/JCI22854 15841182 PMC1070410

[38] Abdoel N Brun S Bracho C Rodriguez MA Blasini AM. Linker for activation of T cells is displaced from lipid rafts and decreases in lupus T cells after activation via the TCR/CD3 pathway. Clin Immunol (2012) 142:243–51. 10.1016/j.clim.2011.12.010 22285373

[39] Cedeno S Cifarelli DF Blasini AM Paris M Placeres F Alonso G Defective activity of ERK-1 and ERK-2 mitogen-activated protein kinases in peripheral blood T lymphocytes from patients with systemic lupus erythematosus: potential role of altered coupling of Ras guanine nucleotide exchange factor hSos to adapter protein Grb2 in lupus T cells. Clin Immunol (2003) 106:41–9. 10.1016/S1521-6616(02)00052-9 12584050

[40] Yi Y McNerney M Datta SK. Regulatory defects in Cbl and mitogen-activated protein kinase (extracellular signal-related kinase) pathways cause persistent hyperexpression of CD40 ligand in human lupus T cells. J Immunol (2000) 165:6627–34. 10.4049/jimmunol.165.11.6627 11086108

[41] Diaz-Gallo LM Sanchez E Ortego-Centeno N Sabio JM Garcia-Hernandez FJ de Ramon E Evidence of new risk genetic factor to systemic lupus erythematosus: the UBASH3A gene. PLoS One (2013) 8:e60646. 10.1371/journal.pone.0060646 23565265 PMC3614928

[42] Katsiari CG Kyttaris VC Juang YT Tsokos GC. Protein phosphatase 2A is a negative regulator of IL-2 production in patients with systemic lupus erythematosus. J Clin Invest (2005) 115:3193–204. 10.1172/JCI24895 16224536 PMC1253625

[43] Tan W Sunahori K Zhao J Deng Y Kaufman KM Kelly JA Association of PPP2CA polymorphisms with systemic lupus erythematosus susceptibility in multiple ethnic groups. Arthritis Rheum (2011) 63:2755–63. 10.1002/art.30452 21590681 PMC3163110

[44] Graham RR Cotsapas C Davies L Hackett R Lessard CJ Leon JM Genetic variants near TNFAIP3 on 6q23 are associated with systemic lupus erythematosus. Nat Genet (2008) 40:1059–61. 10.1038/ng.200 19165918 PMC2772171

[45] Morel L Blenman KR Croker BP Wakeland EK. The major murine systemic lupus erythematosus susceptibility locus, Sle1, is a cluster of functionally related genes. Proc Natl Acad Sci U S A (2001) 98:1787–92. 10.1073/pnas.98.4.1787 11172029 PMC29335

[46] Kyttaris VC Tsokos GC. Targeting lymphocyte signaling pathways as a therapeutic approach to systemic lupus erythematosus. Curr Opin Rheumatol (2011) 23:449–53. 10.1097/BOR.0b013e328349a242 21720246 PMC3158574

[47] Jury EC Flores-Borja F Kalsi HS Lazarus M Isenberg DA Mauri C Abnormal CTLA-4 function in T cells from patients with systemic lupus erythematosus. Eur J Immunol (2010) 40:569–78. 10.1002/eji.200939781 19950182

[48] Lee KM Chuang E Griffin M Khattri R Hong DK Zhang W Molecular basis of T cell inactivation by CTLA-4. Science (1998) 282:2263–6. 10.1126/science.282.5397.2263 9856951

[49] Deng GM Liu L Bahjat FR Pine PR Tsokos GC. Suppression of skin and kidney disease by inhibition of spleen tyrosine kinase in lupus-prone mice. Arthritis Rheum (2010) 62:2086–92. 10.1002/art.27452 20222110 PMC2902591

[50] Ghosh D Tsokos GC Kyttaris VC c-Jun and Ets2 proteins regulate expression of spleen tyrosine kinase in T cells. J Biol Chem (2012) 287:11833–41. 10.1074/jbc.M111.333997 22354960 PMC3320931

[51] Sunahori K Juang YT Kyttaris VC Tsokos GC. Promoter hypomethylation results in increased expression of protein phosphatase 2A in T cells from patients with systemic lupus erythematosus. J Immunol (2011) 186:4508–17. 10.4049/jimmunol.1000340 21346232 PMC3148941

[52] Wertz IE O’Rourke KM Zhou H Eby M Aravind L Seshagiri S De-ubiquitination and ubiquitin ligase domains of A20 downregulate NF-kappaB signalling. Nature (2004) 430:694–9. 10.1038/nature02794 15258597

[53] Josefowicz SZ Lu LF Rudensky AY. Regulatory T cells: mechanisms of differentiation and function. Annu Rev Immunol (2012) 30:531–64. 10.1146/annurev.immunol.25.022106.141623 22224781 PMC6066374

[54] Bonelli M Savitskaya A von Dalwigk K Steiner CW Aletaha D Smolen JS Quantitative and qualitative deficiencies of regulatory T cells in patients with systemic lupus erythematosus (SLE). Int Immunol (2008) 20:861–8. 10.1093/intimm/dxn044 18469329

[55] Miyara M Amoura Z Parizot C Badoual C Dorgham K Trad S Global natural regulatory T cell depletion in active systemic lupus erythematosus. J Immunol (2005) 175:8392–400. 10.4049/jimmunol.175.12.8392 16339581

[56] Langrish CL Chen Y Blumenschein WM Mattson J Basham B Sedgwick JD IL-23 drives a pathogenic T cell population that induces autoimmune inflammation. J Exp Med (2005) 201:233–40. 10.1084/jem.20041257 15657292 PMC2212798

[57] Veldhoen M Hocking RJ Atkins CJ Locksley RM Stockinger B. TGFbeta in the context of an inflammatory cytokine milieu supports de novo differentiation of IL-17-producing T cells. Immunity (2006) 24:179–89. 10.1016/j.immuni.2006.01.001 16473830

[58] Crispin JC Oukka M Bayliss G Cohen RA Van Beek CA Stillman IE Expanded double negative T cells in patients with systemic lupus erythematosus produce IL-17 and infiltrate the kidneys. J Immunol (2008) 181:8761–6. 10.4049/jimmunol.181.12.8761 19050297 PMC2596652

[59] Su D Shen M Li X Sun L Roles of gammadelta T cells in the pathogenesis of autoimmune diseases. Clin Dev Immunol (2013) 2013:985753 10.1155/2013/985753 23533458 PMC3600234

[60] Peng G Wang HY Peng W Kiniwa Y Seo KH Wang RF. Tumor-infiltrating gammadelta T cells suppress T and dendritic cell function via mechanisms controlled by a unique toll-like receptor signaling pathway. Immunity (2007) 27:334–48. 10.1016/j.immuni.2007.05.020 17656116

[61] Li X Kang N Zhang X Dong X Wei W Cui L Generation of human regulatory gammadelta T cells by TCRgammadelta stimulation in the presence of TGF-beta and their involvement in the pathogenesis of systemic lupus erythematosus. J Immunol (2011) 186:6693–700. 10.4049/jimmunol.1002776 21562160

[62] Hsu HC Yang P Wang J Wu Q Myers R Chen J Interleukin 17-producing T helper cells and interleukin 17 orchestrate autoreactive germinal center development in autoimmune BXD2 mice. Nat Immunol (2008) 9:166–75. 10.1038/ni1552 18157131

[63] Wong CK Ho CY Li EK Lam CW. Elevation of proinflammatory cytokine (IL-18, IL-17, IL-12) and Th2 cytokine (IL-4) concentrations in patients with systemic lupus erythematosus. Lupus (2000) 9:589–93. 10.1191/096120300678828703 11035433

[64] Moisan J Grenningloh R Bettelli E Oukka M Ho IC. Ets-1 is a negative regulator of Th17 differentiation. J Exp Med (2007) 204:2825–35. 10.1084/jem.20070994 17967903 PMC2118518

[65] Wong M La Cava A Singh RP Hahn BH. Blockade of programmed death-1 in young (New Zealand Black x New Zealand White)F1 mice promotes the activity of suppressive CD8+ T cells that protect from lupus-like disease. J Immunol (2010) 185:6563–71. 10.4049/jimmunol.0903401 21041733

[66] Chatterjee M Rauen T Kis-Toth K Kyttaris VC Hedrich CM Terhorst C Increased expression of SLAM receptors SLAMF3 and SLAMF6 in systemic lupus erythematosus T lymphocytes promotes Th17 differentiation. J Immunol (2012) 188:1206–12. 10.4049/jimmunol.1102773 22184727 PMC3262878

[67] Chatterjee M Hedrich CM Rauen T Ioannidis C Terhorst C Tsokos GC CD3-T cell receptor co-stimulation through SLAMF3 and SLAMF6 receptors enhances RORgammat recruitment to the IL17A promoter in human T lymphocytes. J Biol Chem (2012) 287:38168–77. 10.1074/jbc.M112.415067 22989874 PMC3488086

[68] Harada T Kyttaris V Li Y Juang YT Wang Y Tsokos GC. Increased expression of STAT3 in SLE T cells contributes to enhanced chemokine-mediated cell migration. Autoimmunity (2007) 40:1–8. 10.1080/08916930601095148 17364491

[69] Edwards LJ Mizui M Kyttaris V. Signal transducer and activator of transcription (STAT) 3 inhibition delays the onset of lupus nephritis in MRL/lpr mice. Clin Immunol (2015) 158:221–30. 10.1016/j.clim.2015.04.004 25869298 PMC4465043

[70] Alvarado-Sanchez B Hernandez-Castro B Portales-Perez D Baranda L Layseca-Espinosa E Abud-Mendoza C Regulatory T cells in patients with systemic lupus erythematosus. J Autoimmun (2006) 27:110–8. 10.1016/j.jaut.2006.06.005 16890406

[71] Vargas-Rojas MI Crispin JC Richaud-Patin Y Alcocer-Varela J. Quantitative and qualitative normal regulatory T cells are not capable of inducing suppression in SLE patients due to T-cell resistance. Lupus (2008) 17:289–94. 10.1177/0961203307088307 18413409

[72] Valencia X Yarboro C Illei G Lipsky PE. Deficient CD4+CD25high T regulatory cell function in patients with active systemic lupus erythematosus. J Immunol (2007) 178:2579–88. 10.4049/jimmunol.178.4.2579 17277168

[73] Hooks JJ Moutsopoulos HM Geis SA Stahl NI Decker JL Notkins AL. Immune interferon in the circulation of patients with autoimmune disease. N Engl J Med (1979) 301:5–8. 10.1056/NEJM197907053010102 449915

[74] Ytterberg SR Schnitzer TJ. Serum interferon levels in patients with systemic lupus erythematosus. Arthritis Rheum (1982) 25:401–6. 10.1002/art.1780250407 6176248

[75] Ronnblom L Potential role of IFNalpha in adult lupus. Arthritis Res Ther (2010) 12:14 10.1186/ar2884 PMC2991776 20392290

[76] Ambrosi A Espinosa A Wahren-Herlenius M. IL-17: a new actor in IFN-driven systemic autoimmune diseases. Eur J Immunol (2012) 42:2274–84. 10.1002/eji.201242653 22949326

[77] Yasuda K Richez C Maciaszek JW Agrawal N Akira S Marshak-Rothstein A Murine dendritic cell type I IFN production induced by human IgG-RNA immune complexes is IFN regulatory factor (IRF)5 and IRF7 dependent and is required for IL-6 production. J Immunol (2007) 178:6876–85. 10.4049/jimmunol.178.11.6876 17513736

[78] Yan B Ye S Chen G Kuang M Shen N Chen S. Dysfunctional CD4+, CD25+ regulatory T cells in untreated active systemic lupus erythematosus secondary to interferon-alpha-producing antigen-presenting cells. Arthritis Rheum (2008) 58:801–12. 10.1002/art.23268 18311820

[79] Bachmann MF Oxenius A. Interleukin 2: from immunostimulation to immunoregulation and back again. EMBO Rep (2007) 8:1142–8. 10.1038/sj.embor.7401099 18059313 PMC2267244

[80] Malek TR. The biology of interleukin-2. Annu Rev Immunol (2008) 26:453–79. 10.1146/annurev.immunol.26.021607.090357 18062768

[81] Sadlack B Lohler J Schorle H Klebb G Haber H Sickel E Generalized autoimmune disease in interleukin-2-deficient mice is triggered by an uncontrolled activation and proliferation of CD4+ T cells. Eur J Immunol (1995) 25:3053–9. 10.1002/eji.1830251111 7489743

[82] Suzuki H Kundig TM Furlonger C Wakeham A Timms E Matsuyama T Deregulated T cell activation and autoimmunity in mice lacking interleukin-2 receptor beta. Science (1995) 268:1472–6. 10.1126/science.7770771 7770771

[83] Fontenot JD Rasmussen JP Gavin MA Rudensky AY. A function for interleukin 2 in Foxp3-expressing regulatory T cells. Nat Immunol (2005) 6:1142–51. 10.1038/ni1263 16227984

[84] Liu Z Davidson A. Taming lupus – a new understanding of pathogenesis is leading to clinical advances. Nat Med (2012) 18:871–82. 10.1038/nm.2752 22674006 PMC3607103

[85] Osorio F LeibundGut-Landmann S Lochner M Lahl K Sparwasser T Eberl G DC activated via dectin-1 convert Treg into IL-17 producers. Eur J Immunol (2008) 38:3274–81. 10.1002/eji.200838950 19039774 PMC2699423

[86] Xu L Kitani A Fuss I Strober W. Cutting edge: regulatory T cells induce CD4+CD25-Foxp3- T cells or are self-induced to become Th17 cells in the absence of exogenous TGF-beta. J Immunol (2007) 178:6725–9. 10.4049/jimmunol.178.11.6725 17513718

[87] Yang XO Nurieva R Martinez GJ Kang HS Chung Y Pappu BP Molecular antagonism and plasticity of regulatory and inflammatory T cell programs. Immunity (2008) 29:44–56. 10.1016/j.immuni.2008.05.007 18585065 PMC2630532

[88] Koenen HJ Smeets RL Vink PM van Rijssen E Boots AM Joosten I. Human CD25highFoxp3pos regulatory T cells differentiate into IL-17-producing cells. Blood (2008) 112:2340–52. 10.1182/blood-2008-01-133967 18617638

[89] Beriou G Costantino CM Ashley CW Yang L Kuchroo VK Baecher-Allan C IL-17-producing human peripheral regulatory T cells retain suppressive function. Blood (2009) 113:4240–9. 10.1182/blood-2008-10-183251 19171879 PMC2676084

[90] Talaat RM Mohamed SF Bassyouni IH Raouf AA. Th1/Th2/Th17/Treg cytokine imbalance in systemic lupus erythematosus (SLE) patients: correlation with disease activity. Cytokine (2015) 72:146–53. 10.1016/j.cyto.2014.12.027 25647269

[91] Yang J Chu Y Yang X Gao D Zhu L Wan L Th17 and natural Treg cell population dynamics in systemic lupus erythematosus. Arthritis Rheum (2009) 60:1472–83. 10.1002/art.24499 19404966

[92] Kleinewietfeld M Manzel A Titze J Kvakan H Yosef N Linker RA Sodium chloride drives autoimmune disease by the induction of pathogenic TH17 cells. Nature (2013) 496:518–22. 10.1038/nature11868 23467095 PMC3746493

[93] Wu C Yosef N Thalhamer T Zhu C Xiao S Kishi Y Induction of pathogenic TH17 cells by inducible salt-sensing kinase SGK1. Nature (2013) 496:513–7. 10.1038/nature11984 23467085 PMC3637879

[94] Mathis KW Venegas-Pont M Masterson CW Wasson KL Ryan MJ. Blood pressure in a hypertensive mouse model of SLE is not salt-sensitive. Am J Physiol Regul Integr Comp Physiol (2011) 301:R1281–5. 10.1152/ajpregu.00386.2011 21917908 PMC3213952

[95] Becker AM Dao KH Han BK Kornu R Lakhanpal S Mobley AB SLE peripheral blood B cell, T cell and myeloid cell transcriptomes display unique profiles and each subset contributes to the interferon signature. PLoS One (2013) 8:e67003. 10.1371/journal.pone.0067003 23826184 PMC3691135

[96] Zou YF Xu JH Tao JH Xu SQ Liu S Chen SY Impact of environmental factors on efficacy of glucocorticoids in Chinese population with systemic lupus erythematosus. Inflammation (2013) 36:1424–30. 10.1007/s10753-013-9682-3 23839650

[97] Weinblatt ME Kavanaugh A Burgos-Vargas R Dikranian AH Medrano-Ramirez G Morales-Torres JL Treatment of rheumatoid arthritis with a Syk kinase inhibitor: a twelve-week, randomized, placebo-controlled trial. Arthritis Rheum (2008) 58:3309–18. 10.1002/art.23992 18975322

[98] Ichinose K Juang YT Crispin JC Kis-Toth K Tsokos GC. Suppression of autoimmunity and organ pathology in lupus-prone mice upon inhibition of calcium/calmodulin-dependent protein kinase type IV. Arthritis Rheum (2011) 63:523–9. 10.1002/art.30085 20954187 PMC3030625

[99] Crispin JC Keenan BT Finnell MD Bermas BL Schur P Massarotti E Expression of CD44 variant isoforms CD44v3 and CD44v6 is increased on T cells from patients with systemic lupus erythematosus and is correlated with disease activity. Arthritis Rheum (2010) 62:1431–7. 10.1002/art.27385 20213807 PMC2879041

[100] Biswas PS Gupta S Chang E Song L Stirzaker RA Liao JK Phosphorylation of IRF4 by ROCK2 regulates IL-17 and IL-21 production and the development of autoimmunity in mice. J Clin Invest (2010) 120:3280–95. 10.1172/JCI42856 20697158 PMC2929726

[101] Ogawa A Andoh A Araki Y Bamba T Fujiyama Y. Neutralization of interleukin-17 aggravates dextran sulfate sodium-induced colitis in mice. Clin Immunol (2004) 110:55–62. 10.1016/j.clim.2003.09.013 14962796

[102] Yi T Zhao D Lin CL Zhang C Chen Y Todorov I Absence of donor Th17 leads to augmented Th1 differentiation and exacerbated acute graft-versus-host disease. Blood (2008) 112:2101–10. 10.1182/blood-2007-12-126987 18596226 PMC2518909

[103] Illei GG Shirota Y Yarboro CH Daruwalla J Tackey E Takada K Tocilizumab in systemic lupus erythematosus: data on safety, preliminary efficacy, and impact on circulating plasma cells from an open-label phase I dosage-escalation study. Arthritis Rheum (2010) 62:542–52. 10.1002/art.27221 20112381 PMC3057537

[104] Solt LA Kumar N Nuhant P Wang Y Lauer JL Liu J Suppression of TH17 differentiation and autoimmunity by a synthetic ROR ligand. Nature (2011) 472:491–4. 10.1038/nature10075 21499262 PMC3148894

